# Fluorine-Tagged 5-Hydroxytryptophan to Investigate Amino Acid Metabolism In Vivo

**DOI:** 10.4061/2010/265069

**Published:** 2010-05-19

**Authors:** Zofia E. Gagnon, Sherry Dingman, Rhys N. Thomas

**Affiliations:** ^1^Marist College, 3399 North Road, Poughkeepsie, NY 12601, USA; ^2^Inovatia Laboratories, LLC, P.O. Box 30, Fayette, MO 65248-0030, USA

## Abstract

Auxin a plant growth hormone, has a metabolic pathway that includes molecules and enzymes like those in animal brains. In this study, tomato plant seedlings (*Lycopersicon esculenta*) were used to investigate the fate of fluorine-tagged 5-hydroxytryptophan (PF-5-HTP) being developed for fluorine spectroscopy and imaging. Seedlings were treated with high or low concentrations of 5-HTP or PF-5-HTP and compared with controls. Metabolites of the PF-5-HTP were quantified using a custom immunoassay for the tag. Serotonin (5-HT) levels were measured with spectrofluorometry and thin-layer chromatography. Plants in treatment conditions had serotonin levels five to six times higher than controls. PF-5-HTP served as a precursor for serotonin in a biosynthetic pathway in this plant model, providing evidence for the bioavailability of the novel molecule. The increase in serotonin in plants grown in media culture supplemented with 5-HTP or PF-5-HTP might have useful applications in pharmacology.

## 1. Introduction

The use of plants as models in pharmacology research could reduce the use of live animals. This approach accords well with Congressional intent and guidelines from the Interagency Coordinating Committee on the Validation of Alternative Methods (ICCVAMs), a permanent committee representing 15 federal regulatory and research agencies that use, generate, and disseminate toxicology testing information. The method described here can be useful in both neuroscience and botany.

In neuroscience, a host of disorders have been linked to the neurotransmitter serotonin. Investigating the serotonin (5-HT) pathways in the brain would be greatly facilitated by having a multiple-fluorine atom tagged version for magnetic resonance spectroscopy (MRS) and functional imaging (fMRI). As serotonin cannot cross the blood brain barrier, it is necessary to produce a tagged version of the precursor that is bioavailable in vivo. One of the metabolic pathways for the biosynthesis of indole-3-acetic acid (auxin) from tryptophan uses the intermediate tryptamine which is structurally like 5-hydroxytryptamine (5-HT). Indole-3-acetic acid (auxin) is an essential hormone for plant growth and development. We used the auxin metabolic pathway as a model for investigating the fate of a novel molecule created for use in brain imaging. 

Toxicologists are interested in auxin for another reason. Having an accurate method for measuring tryptophan derivatives in plant tissues is important because of the role these compounds play in ecosystems and their sensitivity to environmental stress. The complex biochemical systems that regulate the biosynthesis of tryptophan derivatives in plants are dependent on, and sensitive to, multiple factors, including the developmental stage of the plants, local habitat, domestication, and environmental stimuli [1]. To date, investigating the dynamic regulation of such systems has proven difficult because metabolites of interest accumulate only in trace amounts in plants [2]. In general, tryptophan metabolites are of interest for other reasons as well:because of their medicinal properties, presence in nutritious plants, potential toxicity, interactions with endogenous substances, occasional use as illicit psychotropic drugs, and presence in nutritious plants [3]. 

Plants are able to convert tryptophan to indole acetic acid (IAA) by multiple pathways [4]. The pathway in tomatoes (*Lycopersicon esculenta*) uses tryptamine as an intermediary between tryptophan and auxin [5]. Other investigators have quantified the amount of 5-HT in tomato fruit, 3.2 *μ*g g^−1^, and in fresh leaves, 7.5 *μ*g g^−1^ [6, 7]. The quantity of free tryptophan in tomato leaves has been quantified as well, and is about 20 to 40 *μ*g g^−1^ [8]. 

The enzyme tryptophan decarboxylase (TDC) catalyzes the reaction of tryptophan to tryptamine in tomatoes [9]. In the mammalian brain, L-aromatic acid decarboxylase (AADC) catalyzes 5-hydroxytryptophan (5-HTP) to 5-hydroxytryptamine (5-HT) more commonly called serotonin. In animals as well as plants, pyridoxal phosphate serves as a cofactor [10]. Some evidence suggests that the enzyme AADC converts one isomer of perfluoro-tagged 5-hydroxytryptophan (PF-5-HTP) to perfluoro-tagged-5-hydroxytryptamine (PF-5-HT) in vitro [11]. 

**Figure 1:**
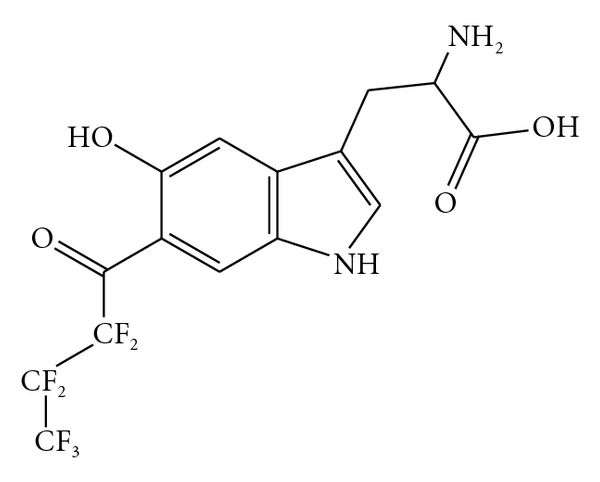
6-heptafluorobutyryl-5-hydroxytryptophan. 5-HTP tagged with a heptafluorobutyryl moiety (perfluorinated 5-hydroxytryptophan, PF-5-HTP). The active isomer of PF-5-HTP has a multiple-fluorine atom tag attached to the 6 position on the indole ring, away from the enzymatic activity.

Given the size of the perfluoro tag in relationship to the molecule to which it is covalently bonded (Figure 1), its bioavailability in vivo is an empirical question. The cuvette-based immunoassay devised by Guo and Thomas to assess the fate of the multiple-fluorine atom tagged monoamine was used to assess the fate of the compound in a preliminary study with plants and in chick embryos [12, 13].

The immunoassay uses an antibody to the multiple fluorine atom tag and, hence, cannot differentiate between tagged 5-HTP and its tagged metabolites. In this study, alkaline butanol column chromatography was used to extract 5-HT from plant tissues. The procedures used for fluoroscopy and thin-layer chromatography (TLC) to compare against the newly developed immunoassay were adapted from those developed by Garcia-Moreno et al. [3].

## 2. Methods

Unless otherwise specified, all chemicals are available from Sigma-Aldrich. ACS-grade chemicals are recommended. The anti-perfluoroalkyl antibodies were developed under an Air Force STTR by the predecessor of Inovatia Laboratories, known as Fayette Environmental Services, Inc. The cell lines are in liquid nitrogen storage at the Cell Immunology Core Facility of the University of Missouri, Columbia. These IgM antibodies are pretreated according to a previously published method [13]. The l-6-heptafluorobutyryl-5-hydroxytryptophan (PF-5-HTP) was synthesized by Inovatia Laboratories for work funded by the National Institute on Drug Abuse. 

### 2.1. In Vitro Plant Culture Experimental Design

The bioavailability of PF-5-HTP was investigated under controlled experimental conditions. Agar-based Murashige and Skoog basal salt mixture (Sigma) contained different concentrations of PF-5-HTP tags (0.3 and 1.7 *μ*g mL^−1^) dissolved in PBS. Separate controls for non-perfluoro-tagged 5-HTP treatments (0.2 and 1.0 *μ*g mL^−1^) in PBS, and PBS only, were used. There were three replicates for each treatment (4 plants per replicate for a total of 12 plants per treatment). The seedlings were grown in Magenta culture vessels containing a total of 50 mL of media with added compounds. The seven treatments were pure media (control), 1 mL PBS, 5 mL PBS, 1 mL PBS containing about 0.2 *μ*g mL^−1^ 5-HTP, 5 mL PBS containing about 5.0 *μ*g mL^−1^ 5-HTP, 1 mL PBS containing PF-5-HTP (biologically equivalent to 0.12 *μ*g 5-HTP) and 5 mL PBS containing PF-5-HTP (biologically equivalent to 3.4 *μ*g 5-HTP.) Plants were maintained during a six-week experimental period in a Lab-Line Growth Chamber at 22.5°C with a 16/8 hr photoperiod under 155 *μ*mol photons m^−2^s^−1^.

### 2.2. Preparation of Tissue Samples for Immunoassay

Tissue samples from each treatment were harvested and separated into leaves and roots. Leaves and roots were cleaned, measured, and weighed before being processed. Plant tissue extract was prepared in 2.0 mL of perchloric acid (0.1 mol L^−1^) using an Omni PCR Tissue Homogenizer at 30,000 rpm. Homogenate was centrifuged for eight minutes. The pH of supernatant was adjusted to 5.7 and homogenate was centrifuged for an additional 12 minutes. The final samples had a volume of about 1.5 mL. The pellets were discarded.

### 2.3. Pretreatment of Methylmethacrylate Cuvettes

Methylmethacrylate cuvettes (4.5 mL) were pretreated by submerging in 2.5 N NaOH in a large beaker containing a large stir bar. The cuvettes were heated overnight at 70°C. During this time the temperature was carefully controlled to prevent cuvette deformation. Cuvettes were then rinsed with distilled water. This process hydrolyzes the methyl esters on the surfaces of cuvettes to produce acid groups. 

### 2.4. Anti-Perfluoroalkyl IgM Treatment

The primary antibody was prepared from an IgM concentrate using a method previously described in detail elsewhere (FES PFC412) [13]. IgM concentrate was added to 15 mL Tris buffer (727 mg *tris*(hydroxymethyl)aminomethane in 15 mL distilled water, adjusted to pH 8 with 1 N HCL). One mL of fresh 2-mercaptoethylamine (2-MEA, 1.2 mg mL^−1^ of distilled water) was then added. The solution was diluted to 20 mL with distilled water, covered, and stirred for one hr at room temperature. During this procedure, the action of the 2-MEA breaks IgM into IgG-like fragments. 

After the one-hour reaction time, the solution was transferred into 14,000 MWCO dialysis tubing (6.4 mL cm^−1^), and the contents dialyzed in a covered 250 mL beaker containing 200 mL PBS/Tween (7.2 pH PBS solution with 0.5 mL Tween 20 per liter). Dialysis was conducted for four hours with two changes of PBS/Tween. The entire content was refrigerated overnight at ~4°C. During this process, small molecules, such as the unreacted MEA and waste fragments, were separated from the antibody concentrate. 

The next day, the contents of the 14,000 MWCO tubing were transferred into 300,000 MWCO tubing (0.32 mL cm^−1^). The tubing, appropriately folded, was placed into a 100 mL graduated cylinder containing 75 mL PBS/Tween and refrigerated overnight. The larger MWCO tubing allows the fragments to diffuse into the cylinder, while the unreacted IgM and the large core ring of reacted IgM (which has no antibody functionality) remain in the tubing.

The following day, the liquid was carefully squeezed from the dialysis tubing into a graduated cylinder, forcing the remaining IgG-like fragments into the cylinder. When less than 2 mL of liquid remained in the tubing, the tube was discarded. The solution in the graduated cylinder was diluted to 100 mL with PBS/Tween and stored in a polypropylene bottle. The concentration of IgG-like antibodies was about 30 ng mL^−1^, assuming 75% recovery.

### 2.5. Immunoassay for Perfluoroalkyl Tags

The procedure for the immunoassay has been previously described in detail [13]. About 1.4 mL of plant extract sample were added to specially prepared cuvettes. The pH of samples and controls was adjusted to a value between 5.5 and 6.0 using 0.25 mol L^−1^ NaOH solution. The final volume was adjusted to 2.0 mL with morpholino ethane sulfonic acid buffer (MES, 0.05 mol L^−1^), prepared from 9.76 g MES in 1 L of water and adjusted to pH 5.5 with 1.0 mol L^−1^ HCl. A cuvette containing only PBS at pH 4.5 served as a negative control. Positive controls were made by adding known quantities of perfluoroalkyl-tagged 5-HTP to PBS at pH 4.5. 

To the stirred cuvettes, 100 *μ*L of N-hydroxysuccinamide and 1-ethly-3-(3-dimethlyaminopropyl) carbodiimide (NHS/EDAC, prepared from 50 mg mL^−1^ NHS and 167 mg mL^−1^ EDAC in MES buffer) were added and stirred for eight hours. NHS/EDAC must be prepared fresh for each batch of samples. Another 100 *μ*L of NHS/EDAC solution were then added and stirring continued for another 8 hours. This process promotes the coupling of amines and acids into amides. The acid groups on the walls of the cuvettes act as anchoring sites for the resulting amino acid chains. Amines in the solution act as terminators to chain growth. 

After the coupling reaction, the cuvettes were rinsed once with distilled water and once with PBS, pH 7.2, without Tween 20. After binding of the analyte to the surfaces of the cuvettes through amide linkages, blocking solution (rabbit gamma globulin at 2 g L^−1^ in PBS, pH 7.2) was added above the mark previously made at the top of the sample solution (~2.5 *μ*L). Stirring was continued for 90 minutes. The cuvettes were then rinsed twice with PBS (pH 7.2) and once with PBS/Tween (pH 7.2). 

To the blocked cuvettes was added serum solution (treated IgM solution to which has been added rabbit gamma globulin at the rate of 1 g L^−1^; shelf life 90 days at 4°C) to a level between the two marks made previously on the cuvettes. This ensures that all regions exposed to the sample are also exposed to antibodies. The extra rabbit gamma globulin is used to reblock regions that may have become uncovered. This solution was stirred 60 minutes in the same manner as for the blocking, using stir bars on the outsides of the cuvettes. After this incubation time, the cuvettes were rinsed three times with PBS/Tween. 

After the primary antibodies are conjugated to the analytes, the secondary antibody solution (goat anti-mouse IgM IgG-HRP, 1 mg L^−1^, plus rabbit gamma globulin, 1 g L^−1^ in PBS/Tween) was added to a height between the marks on the cuvette and stirred for 60 minutes in the same manner as for the blocking and primary antibody. After incubation, the cuvettes were rinsed five times with PBS/Tween. Then PBS (no Tween, pH 4.5) was added and allowed to stand 5 to 10 minutes.

After emptying the cuvette, substrate solution (2 mL, sodium citrate dehydrate, 14.7 g L^−1^; urea hydrogen peroxide, 0.4 g L^−1^; and 2,2-azino-bis(3-ethylbenzthiazoline-6-sulfonic acid), 0.5 g L^−1^ added sequentially to PBS, pH 4.5, with stirring) was added to each cuvette. This pH greatly affects the velocity of the color development, so the final solution should be adjusted to pH 4.5. The solutions were stirred externally 60 minutes. After this period of color development, the solutions were transferred to new, untreated spectrometer cuvettes in order to read the absorbance at 415 nm. The structural material of the final cuvette may be selected for convenience. A standard curve was constructed using known concentrations of PF-5-HTP: 1.875, 3.75, and 7.5 *μ*g mL^−1^. The estimated concentration for PF-5-HTP in the samples was extrapolated from the standard curve. 

### 2.6. Sample Preparation for Column Chromatography

The procedure described below was modified by Garcia-Moreno et al. in [3]. Tomato tissues from each treatment separated into leaf and root groups were homogenized. For every 3 g of homogenate, 20 g of fine sand and 10 g of anhydrous Na_2_SO_4_ were added. After adding 10 mL of alkaline butanol, the mixture of the blended tissue, fine sand, and anhydrous Na_2_SO_4_ was allowed to stand for 30 minutes. Alkaline butanol was prepared using 21 mL of KOH (22% w/w) and 22 mL of isobutanol. The mixture was stirred every five to 10 minutes while let to stand. A glass column was shielded with tin foil prior to packing with the slurry. Once packed, more alkaline butanol was added in 10 mL increments for a total of 40 mL. 

### 2.7. Extraction of Serotonin and Perfluorinated Compounds

Through the column packed with the sand, anhydrous Na_2_SO_4_ and tomato leaf or root mixture was eluted as a drop-by-drop elution of alkaline butanol from the column. The elution was adjusted to last for one hour. 

Separation of serotonin from the alkaline butanol eluate was completed with six washes with 4 mL of deionized water. These washings were followed by a single wash of 4 mL of NaCl_aq_, (0.1336 g of NaCl in 40 mL of distilled water). After adding 2 mL of petroleum ether, the extract was washed with butanol, and the eluate was washed five times with 4 mL 0.1 N HCl. These five 0.1 N HCl extracts were combined, and the final volume-brought to 20 mL. 

### 2.8. Determination of Serotonin Using Fluoroscopy

A calibration curve was made using a prepared solution of serotonin in 0.1 N HCl (1 mg of serotonin in 1 mL 0.1 HCl). From this solution a 10 *μ*g mL^−1^ dilution was made using 0.1 N HCl. Further dilutions of 0.01, 0.02, 0.06, 0.10, 0.40, 0.60, 1.00, 1.50, and 2.00 *μ*g mL^−1^ were made. A calibration curve was then constructed for these concentrations at 295 nm (excitation) and 340 nm (emission) against a 0.1 N HCl blank. The fluorescence data values for the extracted serotonin-HCl solution were recorded at excitation and emission maxima. The serotonin concentrations were extrapolated from the fluorescence values and prepared calibration curve. 

### 2.9. Determination of Serotonin Using TLC

Qualitative identification of serotonin was accomplished using TLC following the method described by Garcia-Moreno et al. in [3]. The serotonin-HCl extract was washed three times, each with 4 mL of ethyl ether. The ether-extracted 0.1 N HCl solution was evaporated to dryness in a rotovapor at 40°C. The resulting crystals were redissolved in 0.2 mL of 0.1 N HCl. Both standard serotonin and the various extracted serotonin samples were spotted on the TLC plate. The samples were developed with a chloroform-methanol-acetic acid solution (70 : 20 : 40). The chromatograph was then sprayed with a fluorogenic detection indicator, O-phthalalldialdehyde (0.5 g in 100 ml ethanol), and subjected to heat (110°C) for 20 minutes. Extracted serotonin yields a brownish-purple color when seen under UV light (360 nm). 

### 2.10. Plant Physiological and Toxicological Responses to Tagged PF-5-HTP

Plant development in response to the different treatments was defined by a number of parameters. A visual observation of physiological and toxicological responses was recorded weekly. Plant height was measured at the end of the experiment. After six weeks, three replicates from each treatment were analyzed for biomass (roots and leaves separately) and physiological effects (chlorophyll content). All plants were examined for typical toxicity symptoms (visual inspection for necrosis, leaf shape and size, and leaf abscission). 

## 3. Results

The plant growth precursor, 5-HTP or its perfluoro-tagged version, PF-5-HTP, was added to plant media in low or high doses (1 mL and 5 mL) and the effect on *L. esculenta* growth and physiology (height, biomass, and chlorophyll content) was investigated. One-way analysis of variance with the Student-Newman-Keuls multiple-comparison test was used to investigate treatment effects on these parameters. The addition of PBS to the growth media had no significant effect on any of the plant growth measures (Table 1). One-way analysis of variance for the treatment effect (5-HTP or PF-5-HTP) at the 1 mL dose (Table 2) resulted in an important difference between conditions for stem length [*F* = 2.87, (df = 2.20), *P* =  .08] and root biomass [*F* = 3.78, (df = 2.12), *P* =  .05] but effects on root length and leaf biomass were weaker at this dose. One-way analysis of variance for the treatment effect at the 5 mL dose (Table 3) resulted in important differences between conditions for stem length [*F* = 3.29, (df = 2.14), *P* =  .07] and root length [*F* = 3.53, (df = 2.14), *P* =  .06]. The effect on biomass [*F* = 7.02, (df = 2.12), *P* =  .01] was significant, while the effect on leaf biomass was much weaker. There were no significant effects on chlorophyll content and no visible symptoms of toxicological response such as chlorosis or changes in plant growth patterns. 

The concentration of the tags detected in tissue samples was extrapolated from a standard curve created using a solution of 1 mg serotonin in 1 mL of 0.1 N HCl. The average concentration of tagged metabolites in leaves was 1.2 *μ*g g^−1^ of fresh weight, and the average concentration was 12 *μ*g g^−1^ of fresh weight in roots. TLC confirmed the presence of serotonin in tissues from various treatments. The concentration of serotonin, extracted from plant material using alkaline butanol, for each treatment was extrapolated from this curve. The estimated serotonin in the 1 mL PF-5-HTP dose treatments was 1.61 *μ*g mL^−1^ for leaves and roots. The estimated serotonin in the 5 mL PF-5-HTP dose treatments was 1.62 *μ*g mL^−1^ for leaves and 1.64 *μ*g mL^−1^ for roots. 

Fluorescence sensitivity units (FSUs) and the corresponding extrapolated concentration values of serotonin in leaves and roots were computed. The results suggest that there was a PF-5-HTP threshold level of about 1 mL of tagged PF-5-HTP per 50 mL of applied growth media. Increasing the PF-5-HTP applications to 5 mL did not increase the amount of serotonin recovered from plant tissues. Serotonin recovery in 1 mL and 5 mL PF-5-HTP treatments was similar (Table 4). This would suggest the existence of a rate-limiting factor in this pathway with saturation achieved below the 1 mL level. The concentration of tag detected in the immunoassay revealed much higher concentrations of tagged compounds in root tissue than in leaves. 

## 4. Discussion

In this study the fate of the PF-tagged monoamines 5-HTP in a metabolic pathway was investigated using a botanical model. The tagged PF-5-HTP was developed for use as an imaging agent for mapping specific neural pathways in animals in vivo. The bioavailability of the L-6-(heptafluorobutyryl)-5-HTP in animals has been investigated using a behavioral paradigm of crayfish aggression [14]. In that study, administering PF-5-HTP mimicked the animal behaviour effect after administration of the untagged version of 5-HTP. Toxicity has not been observed in animal models [15, 16]. Nor was toxicity observed in *L. esculenta *in this study, which demonstrates that plants can serve as useful in vivo models for investigating the biosynthesis of tryptophan metabolites, which are shared by plants and animals. The novel probe can also be used to study the auxin pathway in plants.

In the botanical model described here, the addition of untagged 5-HTP to plant media led to a five-fold increase in serotonin over the amount found in controls. Other investigators have found that transgenic rice overexpressing TDC accumulate serotonin after 5-hydroxytryptophan treatment [17]. The addition of PF- 5-HTP increased the amount of serotonin present in plant tissues sixfold. Given the fact that fewer molecules of the biologically active isomer of PF-5-HTP were available to serve as a substrate for enzymatic conversion to serotonin, this difference is remarkable; and it suggests that the tagged 5-HTP was more biologically active than the endogenous variety. The metabolic pathway used in *L. esculenta* uses the enzyme TDC to convert L-tryptophan into plant growth hormone (IAA) via the intermediate tryptamine. Other studies by Dingman and her colleagues provide evidence the enzyme may favor a specific isomer of the tagged substrate over the endogenous variety, presumably because of its increased polarity [11, 13]. This study provides additional evidence of higher affinity for the PF-tagged variety of 5-HTP than the untagged variety. 

The addition of PF-5-HTP to media did not produce increased growth in plants, although it did increase serotonin recovered from plant tissues. A possible reason for this unusual result may be due to PF-IAA serving as a growth inhibitor in the PF condition. Germinating seeds interact by secreting IAA, with these hormonal interactions producing cluster formation and edge-effect phenomenon [18]. We speculate that seedlings released PF-IAA which, on account of its enhanced polarity, served as an unusually effective suppressor of growth in nearby seedlings. 

The cuvette immunoassay detected about ten times more PF-tagged compounds in plant tissues than the amount of serotonin which could be detected using conventional column chromatography, thinlayer chromatography, or fluoroscopy methods. The antibody used for the immunoassay in this study detects PF tags; it cannot serve to distinguish between PF-5-HTP and its various tagged metabolites. It is possible that only ten percent of the tagged material detected in the immunoassay was PF-5-HT, but it is also possible that the immunoassay was more sensitive than conventional methods. These possibilities are not mutually exclusive. The increase of serotonin in plants grown in media culture supplemented with 5-HTP may have useful pharmaceutical applications [19]. It may also be possible to grow tomato plants for the production of specific metabolites of PF-5-HTP. This would be a novel production method. 

## Figures and Tables

**Table 1:** Growth response of tomato seedlings to treatment with low-and high-dose PBS controls as compared to controls that contain only agar-based Murashige and Skoog basal salt mixture growth media. The enzyme AADC catalyzes the reaction of 5-HTP into 5-HT using the cofactor pyridoxal phosphate. Thus, the PBS controls were needed to determine whether the addition of PBS itself to the media had an effect on plant growth. Numbers in the table represent the mean values ± standard deviation. Each value is based on 3 replicates with 4 plants each (12 per treatment). No statistically significant differences between control and the PBS control treatments were found.

Treatment	Stem length	Root length	Leaf biomass	Root biomass
	(cm)	(cm)	(g)	(g)
Controls	5.0 ± 0.6	5.1 ± 1.1	1.0 ± 0.2	3.7 ± 0.9
1 mL PBS	5.0 ± 1.7	6.4 ± 2.0	1.3 ± 0.5	5.5 ± 2.0
5 mL PBS	5.7 ± 1.9	5.8 ± 2.3	1.0 ± 0.5	4.7 ± 2.2

PBS:phosphate-buffered saline.

**Table 2:** Growth response of tomato seedlings to different low-dose (1 mL) treatments. Low dose of 5-HTP contains ~0.2 *μ*g m*L* ^−1^ 5-HTP in 1 mL PBS. Low dose of PF-5-HTP contains ~0.3 *μ*g PF-5-HTP per m*L* ^−1^ PBS (biologically equivalent to 0.12 *μ*g 5-HTP *μ*g m*L* ^−1^). Numbers in the table represent the mean values ± standard deviation. Each value is based on 3 replicates with 4 plants each (12 per treatment).

Treatment	Stem length (cm)	Root length (cm)	Leaf biomass (g)	Root biomass (g)
1 mL PBS	5.0 ± 1.7	6.4 ± 2.0	1.3 ± 0.5	5.5 ± 2.0^a^
1 mL 5-HTP	6.9 ± 1.7	6.6 ± 1.9	1.7 ± 0.4	8.2 ± 1.7^b^
1 mL PF-5-HTP	4.8 ± 1.7	5.0 ± 1.2	1.1 ± 0.4	6.8 ± 0.8^b^

PBS:phosphate-buffered saline; 5:HTTP:5-hydroxytryptophan; PF:5-HTTP:5-hydroxytryptophan tagged with multiple-fluorine atoms. Values with the same letters (a, b) were not significantly different at probability level *α*< 0.05 as determined by one-way analysis of variance with the Student-Newman-Keuls multiple-comparison test.

**Table 3:** Growth response of tomato seedlings to different high-dose (5 mL) treatments. High dose of 5-HTP contains ~1.0 *μ*g m*L* ^−1^ 5-HTP in 5 mL PBS. High dose of PF-5-HTP contains ~1.7 *μ*g m*L* ^−1^ PF-5-HTP per m*L* ^−1^ PBS (biologically equivalent to 3.4 *μ*g 5-HTP). Numbers in the table represent the mean values ± standard deviation. Each value is based on 3 replicates with 4 plants each (12 per treatment).

Treatment	Stem length (cm)	Root length (cm)	Leaf biomass (g)	Root biomass (g)
5 mL PBS	5.7 ± 1.9^a^	5.8 ± 2.3^a^	1.0 ± 0.5	4.7 ± 2.2^a^
5 mL 5-HTP	5.4 ± 2.0^a^	5.3 ± 1.3^a^	1.1 ± 0.4	7.7 ± 2.2^a^
5 mL PF-5-HTP	3.8 ± 0.7^b^	3.6 ± 1.1^b^	0.6 ± 0.3	4.5 ± 0.8^a^

PBS:phosphate-buffered saline; 5:HTTP:5-hydroxytryptophan; PF-5-HTTP:5-hydroxytryptophan tagged with multiple-fluorine atoms. Values with the same letters (a, b) were not significantly different at probability level *α*< 0.05 as determined by one-way analysis of variance with the Student-Newman-Keuls multiple-comparison test.

**Table 4:** Serotonin concentrations in tomato seedling leaf and root tissues exposed to different treatments of 5-HTP and PF-5-HTP. Numbers in the table represent the mean values ± standard deviation.

Treatment	Serotonin concentration in leaves		Serotonin concentration in roots	
	FSU	*μ*g mL^−1^	FSU	*μ*g mL^−1^
Pure Media	85.3 ± 9.9^a^	0.27 ± 0.03^a^	80.6 ± 21.9^a^	0.27 ± 0.07^a^
1 mL PBS	86.8 ± 23.6^a^	0.28 ± 0.07^a^	81.4 ± 28.9^a^	0.26 ± 0.08^a^
5 mL PBS	63.8 ± 17.4^a^	0.22 ± 0.05^a^	67.4 ± 47.3^a^	0.23 ± 0.18^a^
1 mL 5-HTP	492.1 ± 100.4^b^	1.33 ± 0.28^b^	521.3 ± 106.4^b^	1.41 ± 0.28^b^
5 mL 5-HTP	512.8 ± 149.4^b^	1.38 ± 0.47^b^	563.6 ± 250.7^b^	1.52 ± 0.67^b^
1 mL PF-5-HTP	599.3 ± 174.7^b^	1.61 ± 0.46^b^	600.7 ± 132.7^b^	1.61 ± 0.35^b^
5 mL PF-5-HTP	603.4 ± 91.4^b^	1.62 ± 0.24^b^	611.8 ± 60.7^b^	1.64 ± 0.27^b^

FSU:Fluorescence Sensitivity Units; PBS:phosphate-buffer-saline; 5-HTTP:5-hydroxytryptophan; PF-5-HTTP:5-hydroxytryptophan tagged with multiple-fluorine atoms. Values with the same letters (a, b) were not significantly different at probability level *α*< 0.05 as determined by one-way analysis of variance with the Student-Newman-Keuls multiple-comparison test.
